# Unexpected Response to Nab‐Paclitaxel in Poor Performance Status Lung Adenocarcinoma With a Unique ‘Pan‐Positive’ Tumour Marker Profile: Case Report

**DOI:** 10.1002/rcr2.70442

**Published:** 2025-12-15

**Authors:** Masato Shimozono, Takafumi Kato, Nobuharu Ohshima, Masashi Kitani, Masaomi Maeda, Sumie Nakamura, Kei Kusaka, Masahiro Shimada, Atsuhisa Tamura

**Affiliations:** ^1^ Center for Pulmonary Diseases, NHO Tokyo National Hospital Tokyo Japan; ^2^ Department of Pathology NHO Tokyo National Hospital Tokyo Japan

**Keywords:** case report, nab‐paclitaxel, non‐small cell lung cancer, performance status, tumour marker

## Abstract

Patients with advanced non‐small cell lung cancer and poor performance status (PS) are often excluded from chemotherapy. We present a 63‐year‐old male with metastatic, poorly differentiated lung adenocarcinoma, ECOG PS 3, and an exceptionally broad, markedly elevated panel of tumour markers. He tolerated initial single‐agent nab‐paclitaxel with significant clinical and serological improvement, allowing treatment escalation. This report details the dynamic, often discordant, changes in this marker panel throughout treatment. This case suggests that carefully selected patients with poor PS may derive clinical benefit from chemotherapy and that monitoring a marker profile may provide insights into mechanisms of therapeutic efficacy and resistance.

## Graphical abstract

We report a poor‐PS (ECOG 3) metastatic lung adenocarcinoma patient with an unexpected, favourable response to nab‐paclitaxel, who exhibited a rare ‘pan‐positive’ tumour marker profile with discordant dynamics. This case suggests that selected poor‐PS patients may benefit from chemotherapy, and these marker profiles may reflect tumour heterogeneity and resistance.

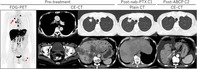

## 1 Introduction

Patients with advanced non‐small cell lung cancer (NSCLC) and a poor performance status (PS) are generally not offered systemic chemotherapy because expected toxicity outweighs benefit [1]. However, this blanket policy may exclude patients who could benefit from individualised therapy. We describe the case of a patient with poor‐PS metastatic lung cancer that challenged standard therapeutic paradigms. The extraordinarily broad and markedly elevated tumour marker profile raised questions about underlying tumour biology and treatment sensitivity, which we explore to underscore the value of tailored decisions even in the most debilitated patient populations.

## 2 Case Report

**FIGURE 1:**
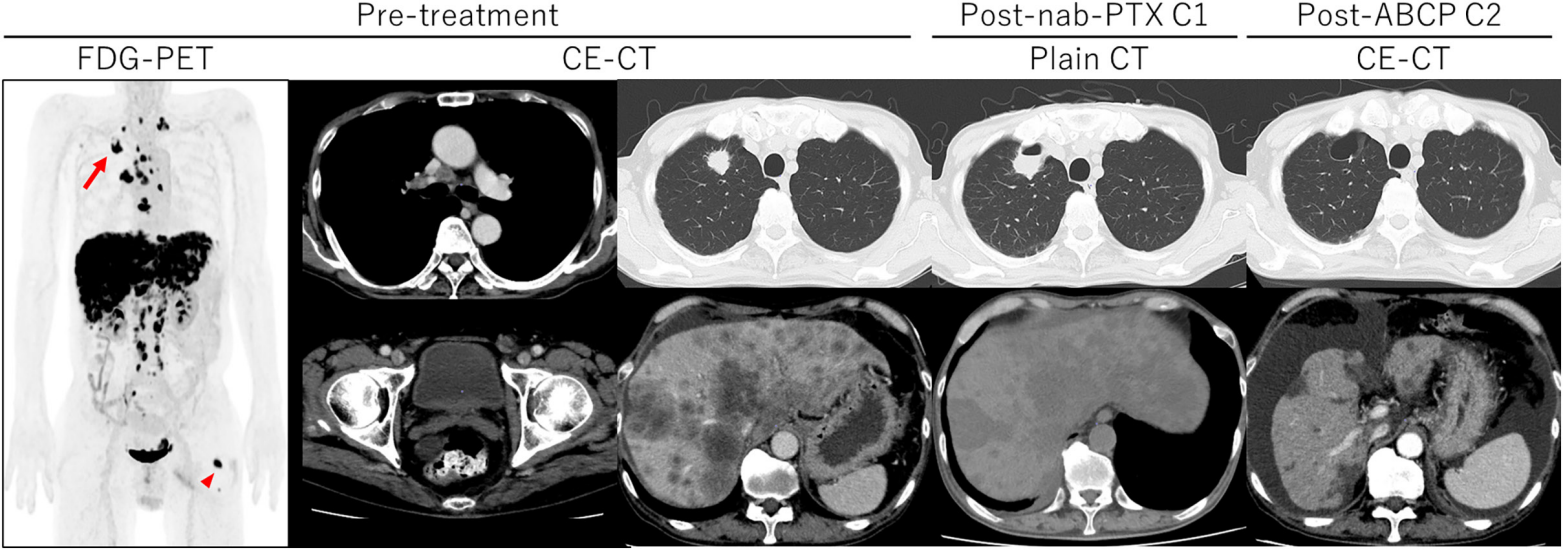
Imaging studies. Pre‐treatment 18F‐FDG‐PET revealed a primary tumour in the right upper lobe (arrow), and extensive liver and nodal metastases. The arrowhead marks a left inguinal lymph node which was subsequently biopsied. After two cycles of ABCP therapy, the primary lung lesion became undetectable, whereas ascites increased.

A 63‐year‐old male smoker presented with weight loss and an Eastern Cooperative Oncology Group (ECOG) PS of 3. Comprehensive imaging with 18F‐FDG‐PET and contrast‐enhanced CT scans revealed a right lung tumour with extensive metastases (liver, bone, peritoneum and widespread lymph nodes), while no other potential primary lesions in the abdomen, including the pancreas or biliary tract, were identified (Figure 1). Laboratory tests showed liver dysfunction and a highly unusual panel of markedly elevated tumour markers (Table 1).

**TABLE 1:** Clinical course by ECOG PS, serological and tumour marker levels.

	Reference range	Pre‐treatment	Post‐ nab‐PTX C1	Post‐ CBDCA+nab‐PTX C1	Post‐ ABCP C1	Post‐ ABCP C3/FN	Pre‐mortem
Day after diagnosis		7	35	69	112	121	149
ECOG PS		3	1	0–1	1	4	4
*Serum markers*							
Alb (g/dL)	4.1–5.1	2.7	2.5	2.8	3	2.3	1.4
T‐Bil (U/L)	0.40–1.50	1.72	0.54	0.86	1.16	3.56	1.91
D‐Bil (U/L)	0.10–0.50	0.97	—	—	—	—	1.59
AST (U/L)	13–30	235	70	50	50	64	106
ALT (U/L)	10–42	132	62	49	27	27	25
LD (U/L)	124–222	620	259	186	373	389	301
ALP (U/L)	38–113	271	180	165	161	82	304
γGT (U/L)	13–64	443	204	186	89	68	247
CRP (mg/dL)	0–0.14	4.08	0.94	1.48	1.33	34.78	2.09
*Tumour markers*							
CEA (ng/mL)	0–5.0	128,517	65,721	12,937	7379	2683	1697
CA19‐9 (U/mL)	0–37.0	1,322,681	344,375	23,853	23,624	4491	7011
CYFRA (ng/mL)	0–2.3	259.9	48.5	14.1	4.3	3.5	4.9
ProGRP (pg/mL)	0–80.9	18.3	—	23.9	36.8	108.2	50.1
NSE (ng/mL)	0–16.3	132	16.6	7.2	12.5	17.5	10.2
SCC (ng/mL)	0–2.5	3.8	1.2	0.6	1.1	18.9	2.6
AFP (ng/mL)	0–10.0	3	3.7	5.4	3.6	—	—
PIVKA‐II (mAU/mL)	0–40	173	97	31	73	—	2308
DUPAN‐2 (U/mL)	0–150	> 1600	1100	280	220	—	58
SPan‐1 (U/mL)	0–30.0	110,000	22,000	2900	2700	—	1100
SLX (U/mL)	0–38	180	> 2000	> 2000	1400	410	310
sIL‐2R (U/mL)	157–474	1210	1560	1000	1570	3850	1280

**FIGURE 2:**
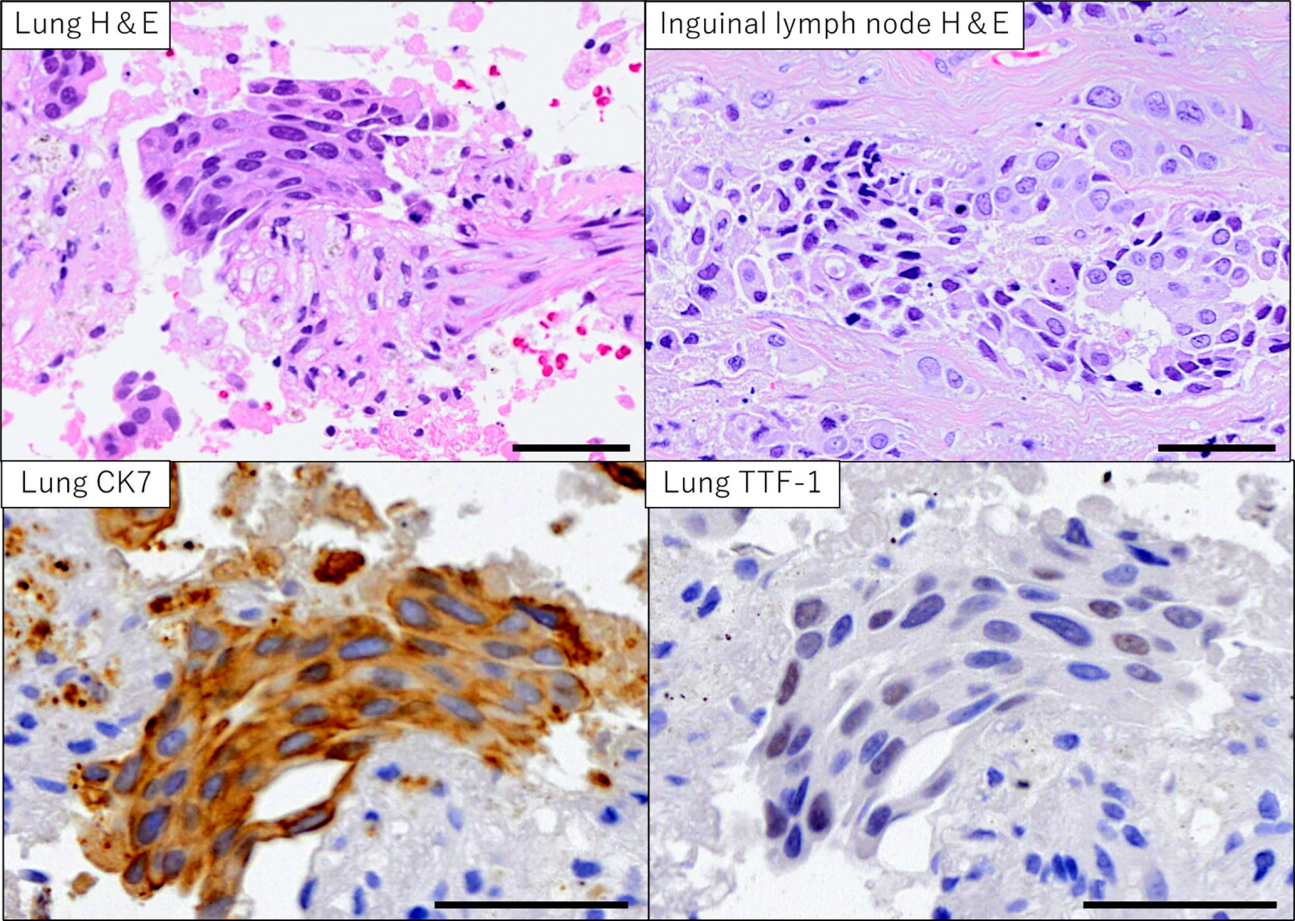
Pathology images. Transbronchial lung biopsy revealed adenocarcinoma, positive for CK7 and weakly positive for TTF‐1. Left inguinal lymph node biopsy exhibited similar findings. Bars = 50 μm.

Transbronchial lung biopsy confirmed poorly differentiated adenocarcinoma, which stained positive for Thyroid Transcription Factor‐1 (TTF‐1). An inguinal lymph node biopsy revealed consistent pathology (Figure 2). Targetable mutations (AmoyDx) and PD‐L1 (22C3/SP263, < 1%) were negative.

Despite the high risk, the patient's strong desire for treatment, along with careful multidisciplinary discussions, led to a cautious trial of single‐agent nab‐paclitaxel (100 mg/m^2^, q3w, d1,8). This resulted in a remarkable clinical improvement (PS improved to 1) and a significant decrease in most tumour markers (Table 1). Treatment was escalated to carboplatin (AUC 4)/nab‐paclitaxel (75 mg/m^2^) (q3w, d1), with further improvement. Subsequent escalation to atezolizumab (1200 mg), bevacizumab (15 mg/kg), carboplatin (AUC 4), and paclitaxel (150 mg/m^2^) (ABCP, q3w, d1) was initiated. Contrast enhanced CT confirmed that the lung primary lesion became undetectable while the ascites increased (Figure 1). After the third ABCP cycle, he developed febrile neutropenia and septic shock (CTCAE Grade 4). Although the infection was properly managed, he passed away due to disease progression 5 months after diagnosis. Serial tumour marker levels were monitored throughout the treatment course (Table 1).

## 3 Discussion

This case presents two key points of discussion: the unexpected therapeutic response in a patient with poor PS, and the profound biological implications of the unique tumour marker profile.

The patient's dramatic initial response to nab‐paclitaxel, despite a PS of 3, challenges the general guideline recommendation against systemic chemotherapy in this population [1]. While this recommendation is based on concerns of high toxicity and limited benefit from older studies, our case adds to limited but important data suggesting that a blanket contraindication may be overly restrictive for carefully selected patients. For instance, a retrospective analysis in 2007 by Leong et al. reported a response rate of 19% in patients with ECOG PS 3 NSCLC treated with single‐agent chemotherapy [2]. In addition, Ikeda et al. reported in a retrospective registry analysis that patients with ECOG PS 3 NSCLC diagnosed in 2012 gained a survival benefit from chemotherapy [3]. It is crucial to recognise that these historical data predate significant advances in oncology, including modern supportive care and the development of better‐tolerated therapeutic agents. The advent of drugs with a more favourable safety profile and efficient supportive drugs may allow for effective treatment with reduced toxicity in this fragile population. Our case, which demonstrates a profound response to nab‐paclitaxel, not only supports the premise that selected patients can benefit but also highlights that current therapeutic tools might make such positive outcomes more achievable than in the past, raising an idea that universal contraindication of chemotherapy for these patients may be overly restrictive.

While treating patients with an ECOG PS of 3‐4 remains a significant challenge, studies in patients with PS 2 have provided a rationale for considering therapy in selected “borderline” populations. For instance, Gajra et al. reported in a single‐arm prospective study that a nab‐paclitaxel‐based regimen was feasible and well‐tolerated in patients with NSCLC and a PS of 2, showing a manageable safety profile and promising efficacy [4]. These provide a clinical bridge for cautiously approaching a patient like ours, whose poor PS was deemed tumour‐related and potentially reversible. Although most prospective trials excluded patients with PS ≥ 3, our experience suggests that nab‐paclitaxel may still provide clinical benefit in selected cases with poor PS. However, clinical course following treatment escalation warrants critical reflection. Although the patient demonstrated a remarkable recovery in PS, the transition to the intensive ABCP regimen resulted in severe adverse events and a subsequent decline in general condition. This outcome suggests that a visible improvement in PS does not necessarily equate to the full restoration of physiological reserve required to tolerate high‐intensity combination therapy. Furthermore, despite the initial response, the patient's overall survival was limited to 5 months, reflecting the generally poor prognosis inherent to this population. Therefore, even when initial chemotherapy yields significant clinical improvement, the decision to escalate to more intensive regimens must be approached with extreme caution, carefully weighing the potential for toxicity against the fragility of the patient's underlying condition.

Another striking feature of this case was the ‘pan‐positive’ serological profile. The extreme elevations of markers for adenocarcinoma (CEA, CA19‐9), neuroendocrine tumours (NSE, ProGRP), squamous carcinoma (CYFRA, SCC), and hepato‐pancreato‐biliary cancer (PIVKA‐II, DUPAN‐2) naturally raised the differential diagnosis of synchronous primary cancers. However, the TTF‐1 positivity of the lung biopsy strongly suggests that the tumour was of lung origin. Further, 18F‐FDG‐PET and contrast‐enhanced CT scans did not reveal any other primary lesions, leading to a definitive diagnosis of metastatic lung adenocarcinoma.

The tumour markers from multiple distinct lineages revealed discordant dynamic changes during treatment. This offers a clear serological snapshot of treatment response and emerging resistance; discordant marker shifts may reflect lineage plasticity [5], although this remains speculative without molecular profiling. Unfortunately, for the current case, the autopsy to further investigate molecular profiles in the primary and metastatic lesions was not possible due to the declined proposal.

In conclusion, we report a metastatic lung adenocarcinoma distinguished by a rare ‘pan‐positive’ serum tumour marker profile and a striking early response to chemotherapy despite an ECOG PS of 3. This case illustrates that carefully individualised treatment can yield meaningful clinical benefit even for patients who would ordinarily be considered ineligible for chemotherapy. Moreover, in primary lung cancer, an exceptionally broad marker profile—and serial monitoring of that panel—can reveal discordant trajectories of lineage‐associated markers. This pattern may represent differential responses of heterogeneous tumour subclones, although this possibility remains speculative without molecular profiling.

## Author Contributions

**Masato Shimozono:** conceptualization, investigation, writing – original draft, writing – review and editing. **Takafumi Kato:** conceptualization, investigation, visualisation, writing – original draft, writing – review and editing, supervision. **Nobuharu Ohshima:** writing – review and editing, supervision. **Masashi Kitani:** investigation, visualisation, writing – review and editing. **Masaomi Maeda:** review and editing. **Sumie Nakamura:** writing – review and editing, supervision. **Kei Kusaka:** writing – review and editing, supervision. **Masahiro Shimada:** writing – review and editing, supervision. **Atsuhisa Tamura:** writing – review and editing, supervision.

## Funding

This study was supported, in part, by the Japan Society of Promotion of Science KAKENHI (grant 22K21371).

## Consent

The authors declare that written informed consent was obtained for the publication of this manuscript and accompanying images using the consent form provided by the Journal.

## Conflicts of Interest

The authors declare no conflicts of interest.

## Data Availability

Data sharing not applicable to this article as no datasets were generated or analysed during the current study.
